# Calretinin Immunoreactivity in the VIIIth Nerve and Inner Ear Endorgans of *Ranid* Frogs

**DOI:** 10.3389/fnins.2021.691962

**Published:** 2021-07-07

**Authors:** Ingrid Reichenberger, Claude J. Caussidier-Dechesne, Hans Straka

**Affiliations:** ^1^Department Biology II, Ludwig-Maximilians-University Munich, Planegg, Germany; ^2^INSERM U254, Université de Montpellier, Montpellier, France

**Keywords:** immunocytochemistry, vestibular, auditory, hair cells, calcium-binding proteins

## Abstract

Calcium-binding proteins are essential for buffering intracellular calcium concentrations, which are critical for regulating cellular processes involved in neuronal computations. One such calcium-binding protein, calretinin, is present in many neurons of the central nervous system as well as those which innervate cranial sensory organs, although often with differential distributions in adjacent cellular elements. Here, we determined the presence and distribution of calretinin-immunoreactivity in the peripheral vestibular and auditory system of *ranid* frogs. Calretinin-immunoreactivity was observed in ganglion cells innervating the basilar and amphibian *papilla*, and in a subpopulation of ganglion cells innervating the saccular epithelium. In contrast, none of the ganglion cells innervating the lagena, the utricle, or the three semicircular canals were calretinin-immunopositive, suggesting that this calcium-binding protein is a marker for auditory but not vestibular afferent fibers in the frog. The absence of calretinin in vestibular ganglion cells corresponds with the lack of type I hair cells in anamniote vertebrates, many of which in amniotes are contacted by the neurites of large, calyx-forming calretinin-immunopositive ganglion cells. In the sensory epithelia of all endorgans, the majority of hair cells were strongly calretinin-immunopositive. Weakly calretinin-immunopositive hair cells were distributed in the intermediate region of the semicircular canal *cristae*, the central part of the saccular *macula*, the utricular, and lagenar striola and the medial part of the amphibian *papilla*. The differential presence of calretinin in the frog vestibular and auditory sensory periphery might reflect a biochemical feature related to firing patterns and frequency bandwidths of self-motion *versus* acoustic stimulus encoding, respectively.

## Introduction

Calcium-binding proteins regulate cellular calcium homeostasis and thus control neuronal processes, such as excitability thresholds, response dynamics, synaptic transmission mechanisms, and indirectly influence neuronal susceptibility to impairments (Baimbridge et al., 1992; Fairless et al., 2019; Schwaller, 2002). In the inner ear, calcium-binding proteins, such as calretinin (CaR), calbindin, parvalbumin, calmodulin, oncomodulin, or the S-100 protein are present in differential, yet partly overlapping subpopulations of hair cells and afferent neurons as demonstrated in a variety of vertebrate species (Saidel et al., 1990; Demêmes et al., 1992; Kerschbaum and Hermann, 1993; Raymond et al., 1993; Roberts, 1993; Jaramillo, 1995; Pack and Slepecky, 1995; Baird et al., 1997; Kevetter and Leonard, 2002a,b; Desai et al., 2005a,b; Simmons et al., 2010; Lysakowski et al., 2011; Hoffman et al., 2018; Prins et al., 2020). The variety of proteins suggests that their different calcium-buffer capacities correlate with the dynamic properties or requirements of a particular cell type (Heizmann and Hunziker, 1991; Baimbridge et al., 1992). Accordingly, the presence of the same calcium-binding protein in different neurons might indicate similar buffer-requirements for comparable neuronal computations, which conveniently render the different proteins suitable as a tag for specific cell groups (Baimbridge et al., 1992).

Calretinin is the most-studied calcium-binding protein in the inner ear (Dechesne et al., 1991, 1993, 1994; Demêmes et al., 1992; Desmadryl and Dechesne, 1992; Raymond et al., 1993; Imamura and Adams, 1996; Edmonds et al., 2000; Kevetter and Leonard, 2002a; Desai et al., 2005a,b; Lysakowski et al., 2011; Holt et al., 2015; Jordan et al., 2015). Immunohistochemical evaluation of the rodent peripheral vestibular system indicated that CaR is present in a morpho-physiologically distinct population of afferents, the calyx fibers (Desmadryl and Dechesne, 1992; Desai et al., 2005a,b; Lysakowski et al., 2011). These calyx fibers, present in all amniote vertebrates, predominate in central epithelial areas and contact type I hair cells, whereas bouton fibers preferentially terminate in peripheral areas on type II hair cells (Goldberg, 2000). Dimorphic fibers supply both type I and type II hair cells throughout most of the epithelia with calyx and bouton endings, respectively (Goldberg, 2000). The different synaptic configurations coincide with several other, interrelated morpho-physiological parameters such as axon diameter, discharge regularity, and response dynamics (Paulin and Hoffman, 2019; Goldberg, 2000). Accordingly, larger fibers, which supply more central epithelial regions, have a more irregular resting discharge and phasic response dynamics; conversely, smaller fibers supply more peripheral regions, have a more regular resting discharge, and exhibit more tonic response properties (Goldberg, 2000).

Anamniote vertebrates lack type I hair cells and thus calyx-type synaptic terminations (Gleisner et al., 1973; Wersäll and Bagger-Sjöback, 1974). Nonetheless, vestibular afferents of, e.g., *ranid* frogs (Honrubia et al., 1981, 1989; Baird and Lewis, 1986; Baird and Schuff, 1994; Reichenberger and Dieringer, 1994; Straka and Dieringer, 2004) form a broad spectrum of fibers with correlations between resting discharge regularity, response dynamics, fiber diameter, and regional innervation of the sensory epithelium similar to the morpho-physiology of vestibular afferents in amniote vertebrates (Goldberg, 2000). This structural and functional similarity prompted us to study the distribution of CaR-immunoreactivity of inner ear neuronal elements in frogs with the hypothesis that CaR has a cell type-specific differential expression pattern.

## Materials and Methods

### Tissue Preparation

Experiments were performed on 19 adult frogs of both sexes (*Rana esculenta* and *Rana temporaria*) and comply with the “Principles of animal care,” publication No. 86-23, revised 1985 of the National Institutes of Health. Permission for these experiments was granted by the Regierung von Oberbayern (211-2531-31/95). In a first set of experiments, animals (*Rana esculenta*, *n* = 7; *Rana temporaria*, *n* = 6) were anesthetized with 0.1% 3-aminobenzoic acid ethyl ester (MS-222) and perfused transcardially with frog Ringer solution (75 mM NaCl, 25 mM NaHCO_3_, 2 mM CaCl_2_, 2 mM KCl, 0.5 mM MgCl_2_, and 11 mM glucose; pH 7.4; 5 ml), followed by 4% paraformaldehyde in 0.1 M phosphate buffer (PB, pH 7.4). The VIIIth nerves along with the otic capsule and all inner ear organs were removed on both sides, post-fixed for 2 h, immersed in 15% sucrose and kept in 30% sucrose overnight at 4°C. The nerves and attached sensory epithelia were cut on a cryostat (10 μm) and sections were mounted directly on glass slides. In a second set of experiments, frogs (*Rana esculenta*, *n* = 6) were anesthetized and decapitated. The inner ears on both sides were quickly removed, dissected and the individual sensory epithelia immersed in 4% paraformaldehyde fixative in 0.1 M PB (pH 7.4) for 3–5 h. Subsequently, the sensory epithelia of two frogs were embedded in 4% agarose in 0.1 M phosphate-buffered saline (PBS, pH 7.4), cut on a vibratome in 50 μm sections, collected and stored in PBS. The sensory epithelia of four other frogs were stored as whole-mounts in PBS until further use.

### Immunocytochemical Procedures

Consecutive cryostat sections mounted on glass slides in the first set of experiments were processed for calretinin immunocytochemistry using the avidin-biotin-complex method. Accordingly, cryostat sections were washed in PBS, pre-incubated for 1 h with PBS containing 0.3% Triton X-100 and 2% normal goat serum and incubated overnight at 4°C with polyclonal rabbit anti-calretinin antibodies (1:5,000, SWant). Thereafter, sections were rinsed in PBS, incubated for 1 h with biotinylated goat anti-rabbit IgG (1:100, Vectastain), rinsed again in PBS and incubated for 1 h in the avidin-biotin complex in PBS (1:100, Vectastain). The peroxidase-labeled avidin-biotin complex was detected by 0.05% diaminobenzidine and 0.01% H_2_O_2_ in 0.05 M *Tris* buffer (pH 7.6). Subsequently, sections were rinsed, dehydrated in alcohol and cover-slipped.

Vibratome sections and whole-mounts of the sensory epithelia in the second set of experiments were processed free-floating to visualize CaR immunofluorescence. Accordingly, the tissue was washed in PBS, pre-incubated for 1 h in PBS containing 0.3% Triton X-100 and 2% normal donkey serum and incubated in anti-calretinin antiserum (1:5,000, SWant). Thereafter, the tissue was washed in PBS and incubated for 3 h in a solution containing Cy3^TM^ -conjugated donkey anti-rabbit IgG (1:500, Jackson ImmunoResearch Laboratories) in darkness. After rinsing in PBS, the vibratome sections were transferred onto glass slides, the whole-mounts on depression slides and cover-slipped with FluorSave reagent (Calbiochem). Primary and secondary antibodies were diluted in the same solution as used for the pre-incubation. No immunostaining was observed in sections processed without primary antibodies. The anti-calretinin antiserum has been characterized by Schwaller et al. (1993) and was shown to react specifically with calretinin in tissue originating from human, monkey, rat, and mouse and not to cross-react with calbindin or other calcium-binding proteins.

### Confocal Microscopy

Visualization of immunofluorescent labeling with confocal microscopy allowed an overview of the different parts of the sensory epithelia in whole-mounts and thick vibratome sections and facilitated the analysis of immunolabeled cellular elements. The immunofluorescence was captured with 0.5–1.0 μm thick single optical sections of tissue using a laser scanning confocal microscope (Bio-Rad, MRC 600). Images were obtained by averaging 4–10 consecutive scans and subsequent processing with the software provided by the manufacturer (Cosmos program). The contrast of the images was optimized using the contrast stretch option. Vibratome sections of 5–26 μm thickness were reconstructed by a projection of serial optical sections.

## Results

### Scarpa’s Ganglion and VIIIth Nerve Afferent Fibers

**FIGURE 1:**
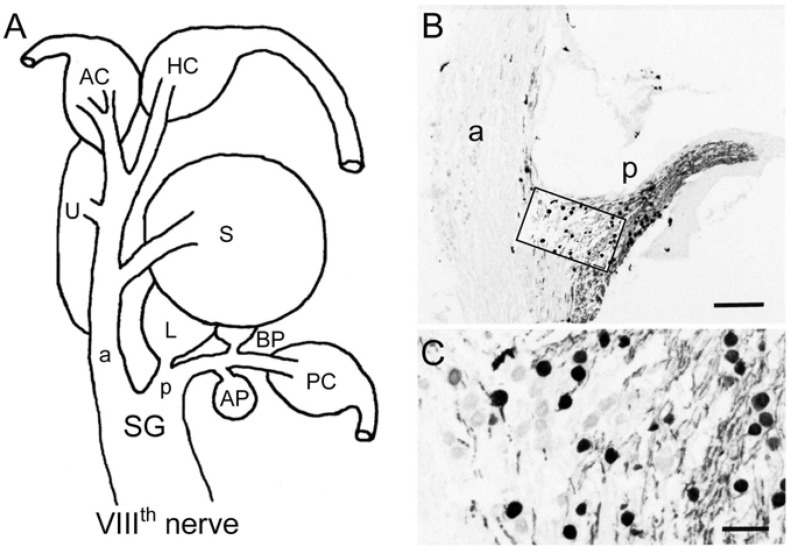
Immunohistochemical localization of calretinin in the VIIIth nerve. **(A)** Schematic of the VIIIth nerve and inner ear endorgans. **(B,C)** Cryostat section through Scarpa’s ganglion (SG) depicting calretinin (CaR)-immunopositive ganglion cells and afferent fibers (peroxidase staining), revealing many cells in the posterior (p) and only few in the anterior branch (a) of the VIIIth nerve; higher magnification **(C)** of the outlined area in panel **(B)**. AP, amphibian *papilla*; AC, HC, PC, anterior, horizontal, posterior semicircular canal; BP, basilar *papilla*; L, lagena; S, saccule; U, utricle. Scale bars are 200 μm in panel **(B)** and 50 μm in panel **(C)**.

The VIIIth nerve contains afferent fibers that connect inner ear endorgans with their target nuclei in the brainstem. In all vertebrate species, the VIIIth nerve ramifies into an anterior and a posterior branch before peripherally entering the otic capsule (a, p in Figure 1A; de Burlet, 1929). In *ranid* frogs, the anterior branch supplies the horizontal and anterior vertical semicircular canal, the utricle and the saccule, while the posterior branch supplies the posterior vertical semicircular canal, the lagena, the basilar and amphibian *papilla* (Figure 1A). The cell bodies of the afferent fibers are located at the junction of the two branches and form Scarpa’s ganglion (SG in Figure 1A). With respect to the presence of calretinin in the VIIIth nerve, strong CaR-immunoreactivity was observed in a subset of ganglion cells and their proximal and distal neurites (Figures 1B,C). CaR-immunopositive cell bodies were relatively small and round with a diameter of ∼15 μm (15.3 ± 3.3 μm; mean ± SD; *n* = 134) and preferentially clustered in the compartment of Scarpa’s ganglion that contains somata and neurites that supply endorgans through the posterior branch. CaR-immunopositive afferent fibers were also observed in the VIIIth nerve between the ganglion and the entrance of the nerve into the brainstem terminating in the dorsal hindbrain known to contain the anuran auditory nuclei (Suarez et al., 1985). However, there were other, small as well as large ganglion cells in the anterior portion of the VIIIth nerve, both of which lacked an obvious CaR-immunoreactivity. These cells, according to their position in the anterior part of the VIIIth nerve supply hair cells in the anterior vertical and horizontal semicircular canal and the utricle, suggesting that size per se does not seem to be a differentiator for CaR-immunoreactivity of the ganglion cells.

The distal neurites of CaR-immunopositive ganglion cells in the posterior branch formed two separate bundles within this branch that could be followed to two endorgans, known to represent anuran auditory organs (Frishkopf and Flock, 1974; Capranica, 1978). One CaR-immunopositive fiber bundle traversed along the posterior branch and curved medially to reach the amphibian *papilla*. The vast majority of fibers innervating this endorgan were intensely CaR-immunopositive with parent cell bodies mainly located in dorsal and lateral portions of the posterior compartment of the ganglion. The second CaR-immunopositive fiber bundle contained only few fibers, diverged from the main branch only very distally to terminate in the basilar *papilla*. In contrast to the amphibian *papilla*, all fibers innervating this auditory endorgan appeared to be CaR-immunopositive. The corresponding cell bodies of afferents innervating the basilar *papilla* were located separately from those of the amphibian *papilla* in the posterior compartment of Scarpa’s ganglion, corroborating earlier observations of an endorgan-specific ganglion cell topography (Hiraoka et al., 1995).

**FIGURE 2:**
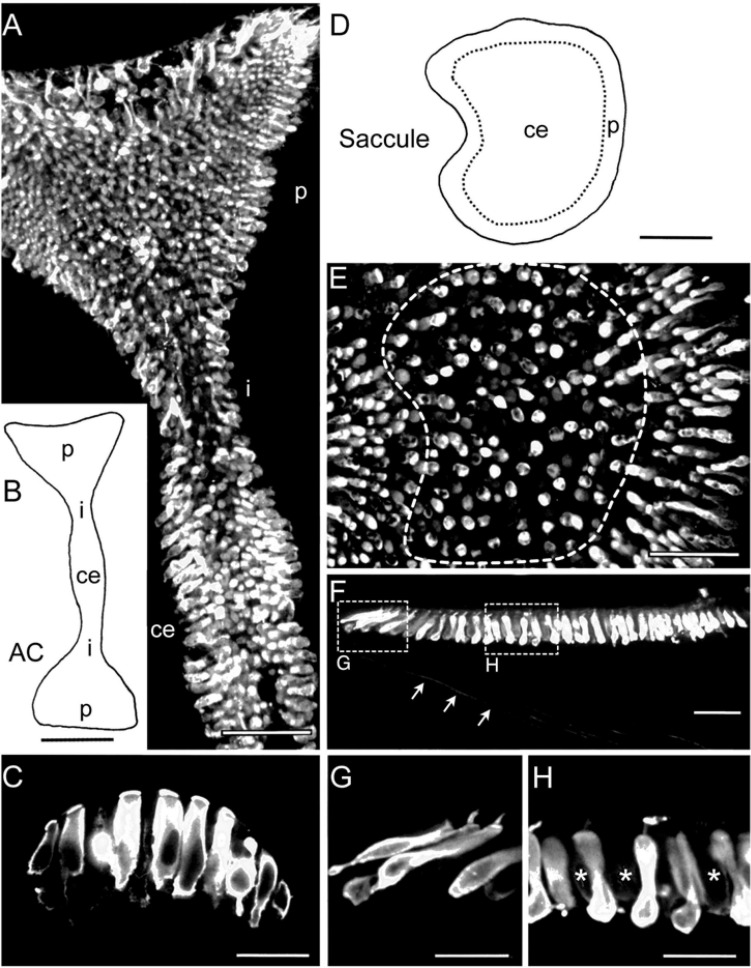
Immunohistochemical localization of calretinin in semicircular canal and saccular sensory epithelia. **(A,B)** Microphotograph of a whole-mount anterior semicircular canal (AC) *crista* **(A)** depicting strongly CaR-immunopositive hair cells with preferential locations in central (ce) and peripheral (p) epithelial regions based on the schematic outline **(B)**. **(C)** Close-up optical reconstruction of a section through the central region of the *crista* illustrating strongly CaR-immunopositive cylindrical hair cells. **(D,E)** Schematic of the saccular *macula* indicating the tentative separation into two epithelial regions (dashed lines) and microphotograph of a whole-mount of the saccule **(E)** depicting strongly CaR-immunopositive hair cells preferentially located in the periphery rather than the center. **(F–H)** Optical reconstruction of a section through the saccule **(F)** depicting a close-up of strongly CaR-immunopositive hair cells in the periphery **(G)** and dumbbell-shaped cells in the center of the epithelium **(H)**; the center contained few interspersed hair cells that were only very weakly CaR-immunopositive (^∗^ in **H**); also note the few CaR-immunopositive afferent fibers (arrows in **F**). i, intermediate region. Scale bars are 50 μm in panels **(A,E,F)**, 200 μm in panels **(B,D)** and 25 μm in panels **(C,G,H)**.

**FIGURE 3:**
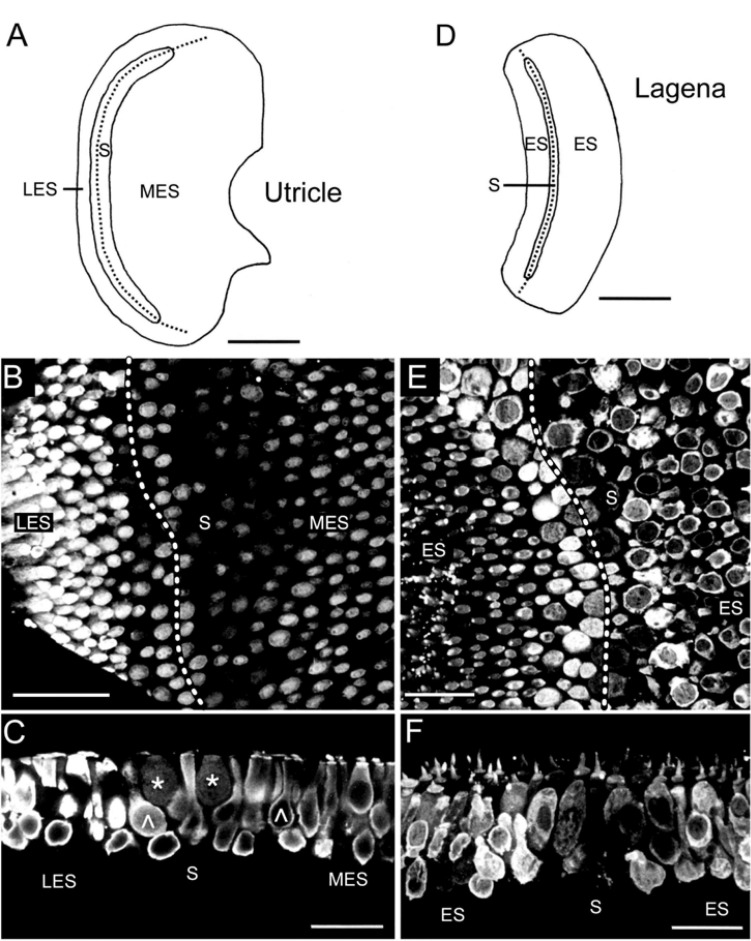
Immunohistochemical localization of calretinin in the utricular and lagenar sensory epithelia. **(A–C)** Schematic of the utricular *macula* indicating the tentative separation into the striola (S) and lateral (LES) and medial extrastriolar (MES) regions and microphotograph of a whole-mount utricle **(B)** with strongly CaR-immunopositive hair cells in the LES and MES and many weakly CaR-immunopositive hair cells along the striola; close-up optical reconstruction of a longitudinal section **(C)** through the utricle shown in panel **(B)**, illustrating several club-like (^∧^) or cylindrical, strongly CaR-immunopositive hair cells and two spherical, weakly CaR-immunopositive hair cells (*). **(D)** Schematic of the lagenar *macula* indicating the tentative separation into a striola (S) and extrastriolar (ES) region. **(E,F)** Optical reconstruction of a horizontal **(E)** and longitudinal section **(F)** through the lagena depicting a region-specific CaR-immunoreactivity with weakly CaR-immunopositive hair cells along the striola; note the intense cilial bundle staining and absence of noticeable CaR-immunoreactivity in supporting cells and nerve fibers. Dotted lines in panels **(A,B,D,E)** indicate the tentative reversal line of hair cell polarization. Scale bars are 200 μm in panels **(A,D)**, 50 μm in panel **(B)** and 25 μm in panels **(C,E,F)**.

The anterior compartment of Scarpa’s ganglion was largely lacking the broad presence of CaR-immunopositive cells, except for a small set of dispersed neurons at the junction of the two ganglionic compartments (Figure 1B). These cells were round and similarly small in diameter as compared to those located in the posterior compartment of Scarpa’s ganglion (15.7 ± 3.5 μm; mean ± SD; *n* = 43). The few CaR-immunopositive fibers projected distally as a small bundle in the anterior branch to reach the saccular *macula* (Figures 1B, 2F). Compared to the entire population of saccular afferents, CaR-immunopositive fibers comprised ∼20–30% of all afferents supplying this endorgan. In contrast, afferent fibers innervating the three semicircular canals (Figures 1B, 2C), the utricle (Figures 1B, 3C), and the lagena (Figure 3F) consistently lacked a noticeable CaR-immunoreactivity. The size of the respective ganglion cells was rather heterogeneous with significantly larger diameters (23.3 ± 7.4 μm; mean ± SD; *n* = 215) as compared to those of the CaR-immunopositive ganglion cells (*p* < 0.0001; Mann-Whitney *U*-test). Collectively, these findings suggest that CaR-immunoreactivity was restricted to afferent fibers of inner ear endorgans involved in the detection of air-borne sound (amphibian and basilar *papilla*, saccule) while afferent fibers of typical vestibular endorgans such as the semicircular canals, utricle, and lagena (Straka and Dieringer, 2004) were devoid of a clear CaR-immunoreactivity. Such distributions thus establish CaR-immunopositivity as a differentiating marker that is able to distinguish frog vestibular and auditory afferents from each other.

### Inner Ear Sensory Epithelia

Beyond afferent immunoreactivity, the sensory epithelia of the different endorgans were tested for CaR-immunoreactivity using either cryostat sections and immunoperoxidase-based antibody detection or whole-mount tissue/vibratome sections and immunofluorescence labeling. Confocal imaging of whole-mounts and vibratome sections revealed different regions of the sensory epithelia, which presented with hair cells of different morphological shape and extent of CaR-immunoreactivity (Figure 2A). The different intensity of CaR-immunopositive hair cells described below is likely related to different concentrations but might be also influenced by endorgan-specific aspects such as tissue thickness, confocal stack size or antibody penetration. In contrast to quantitative studies of concentration and distribution of calcium buffering proteins in the mammalian cochlea (Hackney et al., 2003, 2005), the present approach was purely descriptive and qualitative, providing a general overview of the distribution of CaR in the inner ear of frogs. The confocal images, illustrating CaR-immunofluorescence, are of high contrast rendering even the outline of presumed “CaR-immunonegative” hair cells visible. Accordingly, the presence and tentative absence of immunofluorescent labeling was denoted as strong *versus* weak CaR-immunopositivity.

**FIGURE 4:**
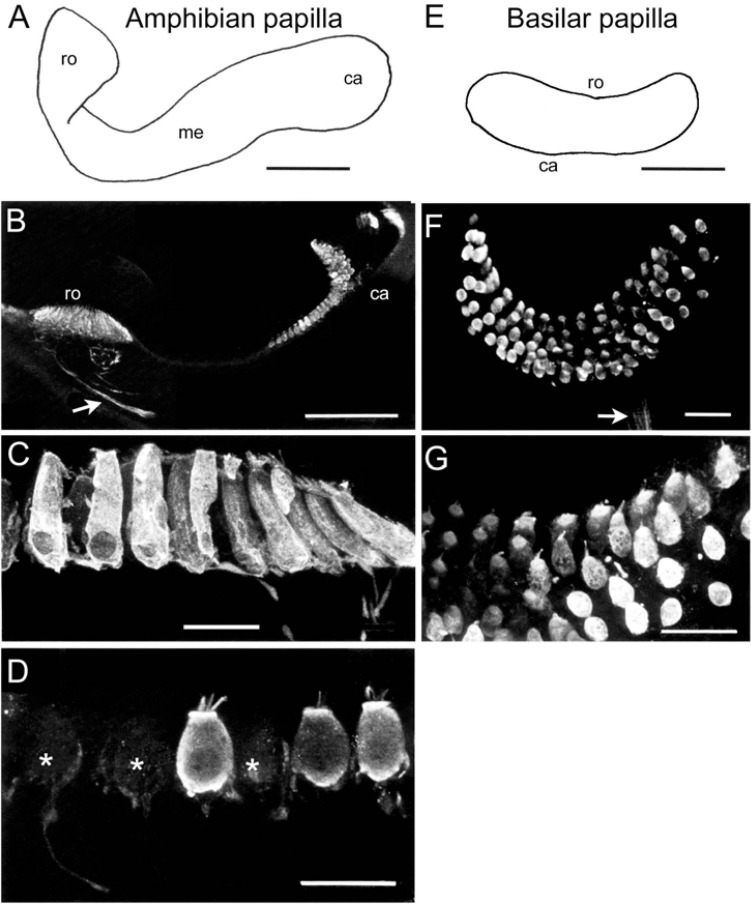
Immunohistochemical localization of calretinin in the sensory epithelium of the amphibian and basilar *papilla*. **(A)** Schematic of the amphibian *papilla* distinguishing a rostral (ro), medial (me), and caudal (ca) region. **(B)** Optical reconstruction of a section through the rostral and caudal end of the amphibian *papilla* depicting strongly CaR-immunopositive hair cells as well as few weakly CaR-immunolabeled afferent fibers (arrow). **(C,D)** Close-up optical reconstructions of cylindrical, strongly CaR-immunopositive hair cells in the rostral region **(C)** and of spherical hair cells in the medial region of the amphibian *papilla* **(D)**, some of which were only very weakly CaR-immunopositive (*). **(E)** Schematic of the basilar *papilla* representing a small, oval-shaped epithelial area. **(F,G)** Microphotographs of a whole-mount basilar *papilla* **(F)** depicting small, spherical and strongly CaR-immunopositive hair cells **(G)** innervated by weakly CaR-immunopositive fibers [arrow in panel **(F)**]. Scale bars are 200 μm in panels **(A,B)**, 50 μm in panel **(E)** and 25 μm in panels **(C,D,F,G)**.

Based on their shapes, hair cells were categorized into four subtypes. (1) Club-like hair cells with a narrow cytoplasmic projection to the epithelial surface and an expanded basal portion with occasional short processes. (2) Cylindrical hair cells with a relatively uniform width from the apex to the base. (3) Spherical hair cells with a larger diameter in the central region compared to the apical or basal portion of the cell body. (4) Dumbbell-shaped hair cells with a smaller diameter in the central region of the cell body than at the apical and basal end of the cell body (present only in the saccule). CaR-immunoreactivity was generally observed in all sensory epithelia (Figures 2–4), however, it was restricted to neurosensory structures, such as hair cells or afferent fibers. Non-neurosensory cell types, such as supporting cells intercalated between hair cells, were at best only very weakly CaR-immunopositive. Intracellularly, the staining of CaR-immunopositive hair cells was particularly strong in the cytoplasm, the cuticular plate, and the cilial bundle, but weak or absent in the nucleus and nucleolus. Given the differences in CaR-immunopositive cell morphologies across endorgans, within-endorgan CaR-immunopositive cell subtypes were further examined.

#### Semicircular Canal *cristae*

The epithelium of the anterior and posterior vertical semicircular canal *cristae* form a symmetric haltere-like shape with two oppositely oriented, but proportional, peripherally enlarged regions and two thin intermediate areas adjacent to a broader central region (Figures 2A,B). The receptor area of the horizontal semicircular canal forms half of a vertical semicircular canal *crista*. Strongly CaR-immunopositive hair cells were found in the *cristae* of all semicircular canals, although with differential regional distributions. Hair cells in central and peripheral regions expressed an intense CaR-immunoreaction, whereas a considerable fraction of hair cells in the intermediate region appeared to express at most a very weak CaR-immunoreaction (Figure 2A). Strongly CaR-immunopositive hair cells were mostly cylindrical (Figure 2C) or club-like, while weakly CaR-immunopositive hair cells in the intermediate region were large, spherical cells.

#### Saccular *macula*

The sensory epithelium of the saccule has the shape of a flattened kidney with the *hilus* indicating the entrance of the nerve branch into the epithelium (Figure 2D). Hair cells in the peripheral part of the saccular *macula* were consistently and strongly CaR-immunopositive, whereas the central part of the *macula* contained a mixture of few, strongly CaR-immunolabeled hair cells intermingled with a substantial number of weakly CaR-immunolabeled hair cells (Figures 2E,F,H). Strongly CaR-immunopositive hair cells in the peripheral part of the *macula* had a club-like shape (Figure 2G) and usually occupied the first 2–6 cell rows along the edge of the epithelium (Figure 2D). The majority of strongly CaR-immunopositive hair cells in the center of the saccular *macula* were dumbbell-shaped with an occasional CaR-immunopositivity of the cilial bundle (Figure 2H).

#### Utricular and Lagenar *macule*

The utricle also has a kidney-shaped appearance, while the lagena forms a broad arc (Figures 3A,D). The *macule* of both organs are, respectively, divided into the striola, a narrow zone of distinctive morphology, and an extrastriolar region (Figures 3A,D). The striola was easily identifiable by a smaller density of hair cells with shorter cilial bundles as compared to those located in the extrastriolar region. The pattern of CaR-immunolabeling was similar for the utricle and the lagena. All extrastriolar and part of the striolar hair cells were strongly CaR-immunopositive (Figures 3B,C,E,F). Hair cells in the lateral extrastriolar region of the utricle and in the small extrastriolar region of the lagena were mostly club-shaped or cylindrical (Figure 3C). Strongly CaR-immunopositive hair cells along the striola were either club-shaped or cylindrical, while the weakly CaR-immunopositive hair cells were mostly spherical (Figure 3C). Based on the position along the striola, the latter cells potentially correspond to pear-shaped hair cells described for the fish utricle (Chang et al., 1992). Hair cells in the medial extrastriolar region of the utricle and the large extrastriolar area of the lagena decreased in size toward the edges. These latter hair cells were cylindrical, club-shaped, or spherical, and consistently expressed a relatively strong CaR-immunoreactivity (Figures 3C,F), matching the large population of striolar (20–40%) and extrastriolar (70–80%) type II CaR-immunopositive hair cells of rodents (Desai et al., 2005b; see also below).

#### Amphibian and Basilar *papilla*

The receptor area of the amphibian *papilla* forms an elongated and three-dimensionally curved epithelium (Figure 4A), covered by a structure that is reminiscent of a tectorial membrane. The three-dimensional complexity of this endorgan along with the relatively large volume of cartilage, encapsulating the duct lumen (Geisler et al., 1964), prevented a confocal reconstruction of the sensory epithelium. The receptor area can be separated into three zones based on hair cell morphology (Lewis and Li, 1975; Simmons et al., 1994). All hair cells in the caudal and rostral portion of the epithelium were strongly CaR-immunopositive (Figures 4B,C), whereas the medial part contained a sizeable number of very weakly labeled CaR-immunopositive hair cells (Figure 4D). Strongly CaR-immunopositive hair cells in the rostral portion were mostly cylindrical or club-shaped (Figure 4C), while those in the caudal portion were spherical and noticeably smaller. The medial portion of the amphibian *papilla* formed a transitory zone with hair cells exhibiting features intermediate to those located in the adjacent rostral and caudal sections of the endorgan, a condition that was largely independent of the overall level of CaR-immunoreactivity (Figure 4D). In compliance with the findings on the CaR-immunoreactivity of *papillar* afferent fibers (see Figure 1), a more or less distinct CaR-immunofluorescent fiber bundle was encountered (arrow in Figure 4B). The poor visibility of the fibers is likely due to the small proportion of optical sections containing immunolabeled afferents within the entire confocal image stack. The basilar *papilla* (Figure 4E) represents a small, oval-shaped, and dorso-ventrally curved epithelium with ∼60 hair cells in ranid frogs (see Geisler et al., 1964). The sensory epithelium was found to consist of 5–6 parallel rows of morphologically rather homogeneous hair cells (Figure 4F) that were surmounted by the tectorial-like membrane. All hair cells were relatively small and of spherical shape and expressed either a strong or at least a moderate CaR-immunoreactivity (Figures 4F,G). Compatible with the strong CaR-immunoreactivity of *papillar* afferents illustrated in Figure 1, small bundles of CaR-immunofluorescent fibers were consistently encountered at the basal aspect of the epithelium (arrow in Figure 4F). The rather faint labeling of these afferents likely derives from an under-representation of the fiber bundle within the confocally reconstructed image stack.

## Discussion

Calretinin-immunohistochemistry revealed a population of small, strongly CaR-immunolabeled ganglion cells with thin axons that innervate the amphibian and basilar *papilla* and the saccular epithelium, all of which are endorgans and epithelial areas known to detect air-borne sound. In contrast, ganglion cells and associated afferents innervating typical vestibular sensory endorgans lacked an obvious CaR-immunoreactivity. In contrast to the selective labeling of afferent fibers, the majority of hair cells in all endorgans were strongly CaR-immunopositive, however, with variations related to epithelial location and cell morphology.

### Morpho-Physiology of Ganglion Cells

The selective CaR-immunopositivity of all *papilla* and a subpopulation of saccular ganglion cells suggests that CaR is a marker for non-vestibular afferent fibers, which transmit sound information (Lewis et al., 1982a,b). In fact, the amphibian and basilar *papillae* represent morpho-physiologically specialized endorgans for the reception of air-borne sound with different frequency ranges, respectively (Frishkopf and Flock, 1974; Feng et al., 1975; Lewis et al., 1982a). While the amphibian *papilla* is sensitive to frequencies of 100–1,000 Hz, the basilar *papilla* detects air-borne sound with frequencies of 1,000–2,000 Hz (Lewis et al., 1982a). The encoding and transmission of these high frequency sensory stimuli (as compared to vestibular stimuli) by afferent neurons of both *papillar* organs requires physiological properties that ensure phase-locked neuronal activity during prolonged episodes of acoustic stimuli as occurring during intraspecies communication (Schwartz and Simmons, 1990). Thus, frog auditory nerve fibers from the *papilla* organs require adaptations that allow a sustenance of phase-locked afferent spikes related to the frequency of the conspecific vocalization patterns (Capranica and Moffat, 1975; Feng et al., 1975). Such a capacity critically depends on specific biochemical properties, including the endowment with calcium-binding proteins (Baimbridge et al., 1992). These calcium-buffers assist in rendering acoustic afferents dynamically capable of persistent spiking that is phase-coupled to the sound stimulus frequency (Ronken, 1991). Vocalization-related acoustic afferent activity would in fact be promoted by the presence of CaR, known to possess a relatively fast kinetics for buffering intracellular Ca^2+^-levels as well as to facilitate efficient Ca^2+^-clearance by a cooperative binding of Ca^2+^ (Barinka and Druga, 2010).

The presence of CaR-immunopositive saccular afferents is consistent with this interpretation given the reported sensitivity of this endorgan also for sound (Ashcroft and Hallpike, 1934; Moffat and Capranica, 1976; Koyama et al., 1982). Saccular fibers are sensitive to a relatively broad range of sound frequencies (Moffat and Capranica, 1976). These sound-sensitive fibers comprise ∼30% of all saccular afferents in toad (Moffat and Capranica, 1976) with similar discharge patterns as fibers that innervate the amphibian and basilar *papillae* (Feng et al., 1975). The fraction of these physiologically identified saccular fibers coincides in magnitude with the 20–30% CaR-immunopositive saccular afferents encountered in the present study, suggesting that the latter indeed represent sound-sensitive saccular afferents. According to this classification scheme, CaR-immunopositivity might be a suitable tag for auditory afferent fibers, consistent with their exclusive central termination in the “dorsal nucleus” of the hindbrain (not shown), known to form the auditory relay area in anurans (Feng and Lin, 1996). This immunohistochemical organization of anuran auditory afferents is consistent with the CaR-immunopositivity of most spiral ganglion cells innervating the mammalian cochlea (Dechesne et al., 1991, 1994; Imamura and Adams, 1996). Therefore, CaR might have evolved as a contributing molecular player toward a necessary physiological pre-requisite that permits encoding of persistent high-frequency acoustic stimuli.

The lack of CaR-immunoreactivity by ganglion cells and associated afferent fibers that innervate the three semicircular canals, the utricle, and the lagena complement the suggestive evidence that CaR is a marker for auditory afferents. While semicircular canals and the utricle have an exclusive vestibular function in almost all vertebrates (Straka and Dieringer, 2004), the frog lagena represents an endorgan with a dual function: detection of tilt and translation and substrate-borne vibrations (MacNaughton and McNally, 1946; Caston et al., 1977; Cortopassi and Lewis, 1996). The absence of CaR-immunoreactivity from lagenar fibers suggests that CaR is not only absent from distinct vestibular afferents but also from seismically-sensitive fibers, which apparently separates the latter from the subgroup of air-borne sound-sensitive saccular or *papillar* afferents. This coincides with the sensitivity of most seismic afferents to lower frequencies (<100 Hz; Cortopassi and Lewis, 1996), while sound-sensitive fibers encode considerably higher frequencies (>500 Hz; Ronken, 1991), potentially rendering CaR in the latter afferents an important biochemical substrate for the transmission of respective spike rates and patterns (see above).

In contrast to the complete lack of CaR-immunoreactivity in frog vestibular afferents, a subpopulation of rather thick mammalian vestibular afferents is densely CaR-immunopositive (Dechesne et al., 1991, 1994; Demêmes et al., 1992; Desmadryl and Dechesne, 1992; Raymond et al., 1993; Kevetter and Leonard, 2002a). These latter fibers form calyces with type I hair cells located at the apex of the semicircular canal *cristae* and the striola of the utricle (Desmadryl and Dechesne, 1992; Dechesne et al., 1994). The involvement of these calyces and associated afferent fibers in the encoding and transmission of high frequency/acceleration head motion signals suggests that CaR in these afferent neurons likely ensures the faithful transmission of phase-timed sensory components (Goldberg, 2000). Although such a requirement also applies to motion detection in frogs, the corresponding population of thick anuran vestibular afferents was found to obviously lack noticeable levels of CaR. The difference between mammals and frogs with respect to CaR-immunoreactivity of vestibular afferents is the absence of type I hair cells and calyx synapses in the latter species. This correlation is supported by the progressive establishment of CaR-immunoreactivity during ontogeny in mice. In fact, the developmental appearance of CaR-immunoreactivity in mammalian vestibular afferents coincides with the functional maturation of calyces in the central zone of the semicircular canal *crista* (Dechesne et al., 1994). This apparent dissociation between hair cell and vestibular afferent CaR-immunopositivity complies with the post-mitotic immunoreactivity for CaR in hair cells but not in afferents in rodents (Zheng and Gao, 1997). The subsequent postnatal permanent loss of CaR-immunopositivity in subsets of hair cells and pure calyx afferents might form part of the electrophysiological maturation process of these vestibular elements (Dechesne et al., 1994; Zheng and Gao, 1997). Even though some aspects of mouse inner ear hair cell/afferent CaR-ontogeny are reminiscent of the pattern present in frog, a distinct recapitulation of phylogenetic principles during development is possible but likely too simplistic.

### Morpho-Physiology of Hair Cells

Although frogs possess only type II hair cells, these mechanoreceptor cells form a rather diverse population with morphological differences related to epithelial location (Wersäll and Bagger-Sjöback, 1974). The differences in hair cell and cilial bundle morphology coincide with adaptations of membrane properties and response dynamics (Lewis and Li, 1975; Baird, 1994a,b; Baird and Schuff, 1994). The diversity of the dynamic tuning of hair cells is likely matched by a differential endowment with calcium-binding proteins (Saidel et al., 1990; Baird et al., 1997; Prins et al., 2020). The present results demonstrate that the CaR-immunoreactivity of hair cells might depend on the shape and regional position within the epithelium. Club-like hair cells, located mainly at the edge of the receptor area, consistently expressed strong CaR-immunopositivity. Cylindrical hair cells were also strongly CaR-immunopositive in the *cristae*, the utricle, lagena and amphibian *papilla*, but only very weakly CaR-immunopositive in the saccule. Smaller spherical hair cells were strongly CaR-immunopositive and present in the extrastriolar regions of the utricle and lagena, in the basilar *papilla* and the caudal region of the amphibian *papilla*. In contrast, larger spherical cells consistently expressed an only very weak CaR-immunoreaction and were present in the striolar region of the utricle and lagena, the intermediate region of the *cristae*, and in the medial part of the amphibian *papilla*. This apparently rather erratic and mosaic-like distribution of strong CaR-immunopositivity in hair cells makes it difficult to extract a consistent picture that might be related to physiological profile, shape, or regional location.

Despite the presence of very weakly CaR-immunopositive hair cells, the majority of frog hair cells were strongly CaR-immunopositive with extensive labeling of the cytoplasm and intense staining of the cuticular plate and cilial bundles (Figures 2–4). This pattern, however, differs from a previous study on the distribution of calcium-binding proteins, including calretinin, in bullfrog (*Rana catesbeiana*) otolith organs (Baird et al., 1997). This latter study demonstrated CaR-immunoreactivity exclusively in cilial bundles of saccular and utricular hair cells, but not in the cytoplasm or other subcellular structures. The CaR-immunopositive cilial bundles were homogeneously distributed across the otolithic epithelia (Baird et al., 1997), without evidence for a hair cell shape-specific presence of this calcium-binding protein. This difference with respect to the current study might be related to the employment of different antibodies or different methodological details or might simply reflect species-specific variations. After all, it is known that even rather closely related ranid species such as *Rana pipiens* (Leopard frog), *Rana esculenta* (Common water frog) and *Rana temporaria* (Grass frog) differ in vestibular hair cell morphologies (Guth et al., 1994; Gioglio et al., 1995) along with eco-physiological adaptations such as sensitivity to substrate vibration, locomotor style and proficiency or vestibulo-ocular reflex organization (Pantle and Dieringer, 1998).

The cell type-specific, strong CaR-immunopositivity in the current study allowed linking these hair cells with epithelial regions that were previously characterized by cilial bundle and/or hair cell morphology (Lewis and Li, 1975; Baird, 1994a; Guth et al., 1994; Gioglio et al., 1995). Accordingly, strongly CaR-immunopositive hair cells were located in central and posterior regions of the *cristae*, and peripheral regions of the *macule*. In the intermediate region of the *cristae* and along the striola, hair cells were only weakly labeled. These latter hair cells, most numerous in the striolar region, are characterized by membrane properties that might depend on calcium-binding proteins other than CaR (Gioglio et al., 1995; Chabbert et al., 1997). In contrast, club-like shaped strongly CaR-immunopositive hair cells along the perimeter of the saccule might represent immature hair cells in the process of being added to the sensory epithelium (Lewis and Li, 1973). Although the epithelial region-specific hair cell morpho-physiology yields a clear correlation between structure and function, the presence of calcium-binding proteins in general and of CaR in particular appears to be less deterministic. The observed CaR distribution pattern is obviously not exclusive to a specific hair cell type but rather representative for a larger functional subgroup with physiological properties that are yet to be determined.

With respect to the CaR-immunoreactivity in the inner ear of other vertebrates, mammals also possess CaR-immunopositive hair cells, although with a differential abundance in different endorgans and hair cell types. In the mammalian cochlea, only inner hair cells were CaR-immunopositive, whereas outer hair cells were CaR-immunonegative (Dechesne et al., 1991, 1994; Pack and Slepecky, 1995; Imamura and Adams, 1996). Adult mammalian vestibular hair cells exhibit a widespread presence of CaR in all inner ear sensory organs, a feature that is only gradually acquired during embryonic development and generally maintained in post-mitotic hair cells (Zheng and Gao, 1997). The abundance of CaR in hair cells has subsequently been confirmed, although with varying distributions, in different endorgans and hair cell types (Desai et al., 2005a,b). According to the latter studies, the largest number of CaR-immunopositive hair cells comprised extrastriolar type II hair cells (70–80%), followed by ∼30% of striolar type II hair cells and <10% type I hair cells (Desai et al., 2005a,b). Along with the specific class of CaR-immunolabeled calyx afferents, CaR thus appears to represent a particular marker for inner ear cellular elements (Dechesne et al., 1991, 1994; Pack and Slepecky, 1995; Imamura and Adams, 1996; Zheng and Gao, 1997; Desai et al., 2005a,b).

## Data Availability Statement

The raw data supporting the conclusions of this article will be made available by the authors, without undue reservation.

## Ethics Statement

The animal study was reviewed and approved by Regierung von Oberbayern (211-2531-31/95).

## Author Contributions

IR performed the experiments and analyzed all data, made the figures, and edited the manuscript. CC-D performed the experiments and edited the manuscript. HS planned the experiments and wrote the manuscript. All authors contributed to the article and approved the submitted version.

## Conflict of Interest

The authors declare that the research was conducted in the absence of any commercial or financial relationships that could be construed as a potential conflict of interest.

## References

[B1] Ashcroft D. W. Hallpike C. S. (1934). On the function of the saccule. *J. Laryngol.* 49 450–460.

[B2] Baimbridge K. G. Celio M. R. Rogers J. H. (1992). Calcium-binding proteins in the nervous system. *Trends Neurosci.* 15 303–309. 10.1016/0166-2236(92)90081-i 1384200

[B3] Baird R. A. (1994a). Comparative transduction mechanisms of hair cells in the bullfrog utriculus. I. Responses to intracellular current. *J. Neurophysiol.* 71 666–684. 10.1152/jn.1994.71.2.666 7909840

[B4] Baird R. A. (1994b). Comparative transduction mechanisms of hair cells in the bullfrog utriculus. II. Sensitivity and response dynamics to hair bundle displacement. *J. Neurophysiol.* 71 685–705. 10.1152/jn.1994.71.2.685 7909841

[B5] Baird R. A. Lewis E. R. (1986). Correspondences between afferent innervation patterns and response dynamics in the bullfrog utricle and lagena. *Brain Res.* 369 48–64. 10.1016/0006-8993(86)90512-3 2870777

[B6] Baird R. A. Schuff N. R. (1994). Peripheral innervation patterns of vestibular nerve afferents in the bullfrog utriculus. *J. Comp. Neurol.* 342 279–298. 10.1002/cne.903420210 8201035

[B7] Baird R. A. Steyger P. S. Schuff N. R. (1997). Intracellular distribution and putative functions of calcium-binding proteins in the bullfrog vestibular otolith organs. *Hear. Res.* 103 85–100. 10.1016/s0378-5955(96)00167-0 9007577

[B8] Barinka F. Druga R. (2010). Calretinin expression in the mammalian neocortex: a review. *Physiol. Res.* 59 665–677. 10.33549/physiolres.931930 20406030

[B9] Capranica R. R. (1978). Auditory processing in anurans. *Fed. Proc.* 37 2324–2328. 354968

[B10] Capranica R. R. Moffat A. J. M. (1975). Selectivity of the peripheral auditory system of spadefoot toads (*Scaphiopus couchi*) for sounds of biological significance. *J. Comp. Physiol. A* 100 231–249. 10.1007/bf00614533

[B11] Caston J. Precht W. Blanks R. H. I. (1977). Response characteristics of frog‘s lagena afferents to natural stimulation. *J. Comp. Physiol. A* 118 273–289. 10.1007/bf00614351

[B12] Chabbert C. Chambard J. M. Valmier J. Sans A. Desmadryl G. (1997). Voltage-activated sodium currents in acutely isolated mouse vestibular ganglion neurons. *Neuroreport* 8 1253–1256. 10.1097/00001756-199703240-00039 9175124

[B13] Chang J. S. Popper A. N. Saidel W. M. (1992). Heterogeneity of sensory hair cells in a fish ear. *J. Comp. Neurol.* 324 621–640. 10.1002/cne.903240413 1430341

[B14] Cortopassi K. A. Lewis E. R. (1996). High-frequency tuning properties of bullfrog lagenar vestibular afferent fibers. *J. Vestib. Res.* 6 105–119. 10.3233/ves-1996-6205 8925113

[B15] de Burlet H. M. (1929). Zur vergleichenden anatomie der labyrinthinnervation. *J. Comp. Neurol.* 47 155–169. 10.1002/cne.900470202

[B16] Dechesne C. D. Rabejac D. Desmadryl G. (1994). Development of calretinin immunoreactivity in the mouse inner ear. *J. Comp. Neurol.* 346 517–529. 10.1002/cne.903460405 7983242

[B17] Dechesne C. D. Winsky L. Kim H. N. Goping G. Vu T. D. Wenthold R. J. (1991). Identification and ultrastructural localization of a calretinin-like calcium-binding protein (protein 10) in the guinea pig and rat inner ear. *Brain Res.* 560 139–148. 10.1016/0006-8993(91)91224-o 1722130

[B18] Dechesne C. D. Winsky L. Moniot B. Raymond J. (1993). Localization of calretinin mRNA in rat and guinea pig inner ear by *in situ* hybridization using radioactive and non-radioactive probes. *Hear. Res.* 69 91–97. 10.1016/0378-5955(93)90096-j 8226353

[B19] Demêmes D. Raymond J. Atger P. Grill C. Winsky L. Dechesne C. J. (1992). Identification of neuron subpopulations in the rat vestibular ganglion by calbindin-D 28K, calretinin and neurofilament proteins immunoreactivity. *Brain Res.* 582 168–172. 10.1016/0006-8993(92)90334-6 1498680

[B20] Desai S. S. Ali H. Lysakowski A. (2005a). Comparative morphology of rodent vestibular periphery. II. Cristae ampullares. *J. Neurophysiol.* 93 267–280. 10.1152/jn.00747.2003 15240768 PMC12513555

[B21] Desai S. S. Zeh C. Lysakowski A. (2005b). Comparative morphology of rodent vestibular periphery. I. Saccular and utricular maculae. *J. Neurophysiol.* 93 251–266. 10.1152/jn.00746.2003 15240767 PMC12456082

[B22] Desmadryl G. Dechesne C. J. (1992). Calretinin immunoreactivity in chinchilla and guinea pig vestibular end organs characterizes the calyx unit subpopulation. *Exp. Brain Res.* 89 105–108. 1601088 10.1007/BF00229006

[B23] Edmonds B. Reyes R. Schwaller B. Roberts W. M. (2000). Calretinin modifies presynaptic calcium signaling in frog saccular hair cells. *Nat. Neurosci.* 3 786–790. 10.1038/77687 10903571

[B24] Fairless R. Williams S. K. Diem R. (2019). Calcium-binding proteins as determinants of central nervous system neuronal vulnerability to disease. *Int. J. Mol. Sci.* 20:2146. 10.3390/ijms20092146 31052285 PMC6539299

[B25] Feng A. S. Lin W. Y. (1996). Neural architecture of the dorsal nucleus (cochlear nucleus) of the frog, *Rana pipiens pipiens*. *J. Comp. Neurol.* 366 320–334. 10.1002/(sici)1096-9861(19960304)366:2<320::aid-cne10>3.0.co;2-t 8698890

[B26] Feng A. S. Narins P. M. Capranica R. R. (1975). Three populations of primary auditory fibers in the bullfrog (*Rana catesbeiana*): their peripheral origin and frequency sensitivities. *J. Comp. Physiol. A* 100 221–229. 10.1007/bf00614532

[B27] Frishkopf L. S. Flock A. (1974). Ultrastructure of the basilar papilla, an auditory organ in the bullfrog. *Acta Otolaryngol.* 77 176–184. 10.3109/00016487409124615 4594555

[B28] Geisler C. D. van Bergeijk W. A. Frishkopf L. S. (1964). The inner ear of the bullfrog. *J. Morphol.* 114 43–57. 10.1002/jmor.1051140103 14114962

[B29] Gioglio L. Congiu T. Quacci D. Prigioni I. (1995). Morphological features of different regions in frog crista ampullaris (*Rana esculenta*). *Arch. Histol. Cytol.* 58 1–16. 10.1679/aohc.58.1 7612357

[B30] Gleisner L. Flock Å Wersäll J. (1973). The ultrastructure of the afferent synapse on hair cells in the frog labyrinth. *Acta Otolaryngol.* 76 199–207. 10.3109/00016487309121500 4543915

[B31] Goldberg J. M. (2000). Afferent diversity and the organization of central vestibular pathways. *Exp. Brain Res.* 130 277–297. 10.1007/s002210050033 10706428 PMC3731078

[B32] Guth P. S. Fermin C. D. Pantoja M. Edwards R. Norris C. (1994). Hair cells of different shapes and their placement along the frog crista ampullaris. *Hear. Res.* 73 109–115. 10.1016/0378-5955(94)90288-7 8157499

[B33] Hackney C. M. Mahendrasingam S. Jones E. M. Fettiplace R. (2003). The distribution of calcium buffering proteins in the turtle cochlea. *J. Neurosci.* 23 4577–4589. 10.1523/jneurosci.23-11-04577.2003 12805298 PMC6740801

[B34] Hackney C. M. Mahendrasingam S. Penn A. Fettiplace R. (2005). The concentrations of calcium buffering proteins in mammalian cochlear hair cells. *J. Neurosci.* 25 7867–7875. 10.1523/jneurosci.1196-05.2005 16120789 PMC6725244

[B35] Heizmann C. W. Hunziker W. (1991). Intracellular calcium-binding proteins: more sites than insights. *Trends Biochem. Sci.* 16 98–103. 10.1016/0968-0004(91)90041-s 2058003

[B36] Hiraoka I. Suzuki M. Harada V. Tagashira N. Takumida M. (1995). Anatomical and physiological characteristics of the vestibular ganglion of the bull frog. *Acta Otolaryngol. Suppl.* 519 253–256. 10.3109/00016489509121917 7610880

[B37] Hoffman L. F. Choy K. R. Sultemeier D. R. Simmons D. D. (2018). Oncomodulin expression reveals new insights into the cellular organization of the murine utricle striola. *J. Assoc. Res. Otolaryngol.* 19 33–51. 10.1007/s10162-017-0652-6 29318409 PMC5783930

[B38] Holt J. C. Kewin K. Jordan P. M. Cameron P. Klapczynski M. McIntosh J. M. (2015). Pharmacologically distinct nicotinic acetylcholine receptors drive efferent-mediated excitation in calyx-bearing vestibular afferents. *J. Neurosci.* 35 3625–3643. 10.1523/jneurosci.3388-14.2015 25716861 PMC4339364

[B39] Honrubia V. Hoffmann L. F. Sitko S. Schwartz I. R. (1989). Anatomic and physiological correlates in bullfrog vestibular nerve. *J. Neurophysiol.* 61 688–701. 10.1152/jn.1989.61.4.688 2786056

[B40] Honrubia V. Sitko S. Kimm J. Betts W. Schwartz I. R. (1981). Physiological and anatomical characteristics of primary vestibular afferent neurons in the bullfrog. *Int. J. Neurosci.* 15 197–206. 10.3109/00207458108985857 6172398

[B41] Imamura S. Adams J. C. (1996). Immunolocalization of peptide 19 and other calcium-binding proteins in the guinea pig cochlea. *Anat. Embryol.* 194 407–418. 10.1007/BF00198543 8896705

[B42] Jaramillo F. (1995). Signal transduction in hair cells and its regulation by calcium. *Neuron* 15 1227–1230. 10.1016/0896-6273(95)90003-9 8845148

[B43] Jordan P. M. Fettis M. Holt J. C. (2015). Efferent innervation of turtle semicircular canal cristae: comparisons with bird and mouse. *J. Comp. Neurol.* 523 1258–1280. 10.1002/cne.23738 25560461 PMC4390460

[B44] Kerschbaum H. H. Hermann A. (1993). Calcium-binding proteins in the inner ear of *Xenopus laevis* (Daudin). *Brain Res.* 617 43–49. 10.1016/0006-8993(93)90610-y 8374743

[B45] Kevetter G. A. Leonard R. B. (2002a). Molecular probes of the vestibular nerve. I. Peripheral termination patterns of calretinin, calbindin and peripherin containing fibers. *Brain Res.* 928 8–17. 10.1016/s0006-8993(01)03268-1 11844467

[B46] Kevetter G. A. Leonard R. B. (2002b). Molecular probes of the vestibular nerve. II. Characterization of neurons in Scarpa‘s ganglion to determine separate populations within the nerve. *Brain Res.* 928 18–29. 10.1016/s0006-8993(01)03264-4 11844468

[B47] Koyama H. Lewis E. R. Leverenz E. L. Baird R. A. (1982). Acute seismic sensitivity in the bullfrog ear. *Brain Res.* 250 168–172. 10.1016/0006-8993(82)90964-7 6982744

[B48] Lewis E. R. Li C. W. (1973). Evidence concerning the morphogenesis of saccular receptors in the bullfrog (*Rana catesbeiana*). *J. Morph.* 139 351–362. 10.1002/jmor.1051390305 4539709

[B49] Lewis E. R. Li C. W. (1975). Hair cell types and distributions in the otolithic and auditory organs of the bullfrog. *Brain Res.* 83 35–50. 10.1016/0006-8993(75)90856-2

[B50] Lewis E. R. Baird R. A. Leverenz E. L. Koyama H. (1982a). Inner ear: dye injection reveals peripheral origins of specific sensitivities. *Science* 215 1641–1643. 10.1126/science.6978525 6978525

[B51] Lewis E. R. Leverenz E. L. Koyama H. (1982b). The tonotopic organization of the bullfrog amphibian papilla, an auditory organ lacking a basilar membrane. *J. Comp. Physiol. A* 145 437–445. 10.1007/bf00612809

[B52] Lysakowski A. Gaboyard-Niay S. Calin-Jageman I. Chatlani S. Price S. D. Eatock R. A. (2011). Molecular microdomains in a sensory terminal, the vestibular calyx ending. *J. Neurosci.* 31 10101–10114. 10.1523/jneurosci.0521-11.2011 21734302 PMC3276652

[B53] MacNaughton I. P. McNally W. J. (1946). Some experiments which indicate that the frog‘s lagena has an equilibrial function. *J. Laryngol. Otol.* 61 204–214. 10.1017/s0022215100007842 20282085

[B54] Moffat A. J. Capranica R. R. (1976). Auditory sensitivity of the saccule in the American toad (*Bufo americanus*). *J. Comp. Physiol. A* 105 1–8. 10.1007/bf01380048

[B55] Pack A. K. Slepecky N. B. (1995). Cytoskeletal and calcium-binding proteins in the mammalian organ of Corti: cell type-specific proteins displaying longitudinal and radial gradients. *Hear. Res.* 91 119–135. 10.1016/0378-5955(95)00173-5 8647714

[B56] Pantle C. Dieringer N. (1998). Spatial transformation of semicircular canal signals into abducens motor signals. A comparison between grass frogs and water frogs. *J. Comp. Physiol. A* 182 475–487. 10.1007/s003590050195 9530837

[B57] Paulin M. G. Hoffman L. F. (2019). Models of vestibular semicircular canal afferent neuron firing activity. *J. Neurophysiol.* 122 2548–2567. 10.1152/jn.00087.2019 31693427 PMC6966309

[B58] Prins T. J. Myers Z. A. Saldate J. J. Hoffman L. F. (2020). Calbindin expression in adult vestibular epithelia. *J. Comp. Physiol. A* 206 623–637. 10.1007/s00359-020-01418-6 32350587

[B59] Raymond J. Dechesne C. J. Desmadryl G. Demêmes D. (1993). Different calcium-binding proteins identify subpopulations of vestibular ganglion neurons in the rat. *Acta Otolaryngol.* 503 114–118. 10.3109/00016489309128090 8385864

[B60] Reichenberger I. Dieringer N. (1994). Size-related colocalization of glycine and glutamate immunoreactivity in frog and rat vestibular afferents. *J. Comp. Neurol.* 349 603–614. 10.1002/cne.903490408 7860791

[B61] Roberts W. M. (1993). Spatial calcium buffering in saccular hair cells. *Nature* 363 74–76. 10.1038/363074a0 8479539

[B62] Ronken D. A. (1991). Spike discharge properties that are related to the characteristic frequency of single units in the frog auditory nerve. *J. Acoust. Soc. Am.* 90 2428–2440. 10.1121/1.402047 1663526

[B63] Saidel W. M. Presson J. C. Chang J. S. (1990). S-100 immunoreactivity identifies a subset of hair cells in the utricle and saccule of a fish. *Hear. Res.* 47 139–146. 10.1016/0378-5955(90)90171-k 2228790

[B64] Schwaller B. (2002). ‘New’ functions for ‘old’ proteins: the role of the calcium-binding proteins calbindin D-28k, calretinin and parvalbumin, in cerebellar physiology. Studies with knockout mice. *Cerebellum* 1 241–258. 10.1080/147342202320883551 12879963

[B65] Schwaller B. Buchwald P. Blümcke I. Celio M. R. Hunziker W. (1993). Characterization of a polyclonal antiserum against the purified human recombinant calcium-binding protein calretinin. *Cell Calcium* 14 639–648. 10.1016/0143-4160(93)90089-o 8242719

[B66] Schwartz J. J. Simmons A. M. (1990). Encoding of a spectrally-complex communication sound in the bullfrog‘s auditory nerve. *J. Comp. Physiol. A* 166 489–499. 2332839 10.1007/BF00192019

[B67] Simmons D. D. Bertolotto C. Narins P. M. (1994). Morphological gradients in sensory hair cells of the amphibian papilla of the frog, *Rana pipiens pipiens*. *Hear. Res.* 80 71–78. 10.1016/0378-5955(94)90010-8 7852205

[B68] Simmons D. D. Tong B. Schrader A. D. Hawkes A. J. (2010). Oncomodulin identifies different hair cell types in the mammalian inner ear. *J. Comp. Neurol.* 518 3785–3802. 10.1002/cne.22424 20653034 PMC2909616

[B69] Straka H. Dieringer N. (2004). Basic organization principles of the VOR: lessons from frogs. *Prog. Neurobiol.* 73 259–309. 10.1016/j.pneurobio.2004.05.003 15261395

[B70] Suarez C. Kuruvilla A. Sitko S. Schwartz I. R. Honrubia V. (1985). Central projections of primary vestibular fibers in the bullfrog. II. Nerve branches from individual receptors. *Laryngoscope* 95 1238–1250. 3876498 10.1288/00005537-198510000-00018

[B71] Wersäll J. Bagger-Sjöback D. (1974). “Morphology of the vestibular sense organ,” in *Handbook of Sensory Physiology. Vestibular system. Basic Mechanisms*, ed. Kornhuber H. H. (New York: Springer), 123–170. 10.1007/978-3-642-65942-3_4

[B72] Zheng J. L. Gao W.-Q. (1997). Analysis of rat vestibular hair cell development and regeneration using calretinin as an early marker. *J. Neurosci.* 17 8270–8282. 10.1523/JNEUROSCI.17-21-08270.1997 9334402 PMC6573764

